# A microbiologist’s field guide to community ecology

**DOI:** 10.1093/ismeco/ycag118

**Published:** 2026-05-07

**Authors:** Luca Beldi, Alyssa Henderson, Megan N Y Lee, Julien S Luneau, Florent Mazel, Thomas E Miller, Prajwal Padmanabha, Benjamin Raach, Simon van Vliet, Anna S Weiss, Oliver J Meacock, Margaret A Vogel

**Affiliations:** Institute for Infectious Diseases, University of Bern, Bern, 3001, Switzerland; Department of Environmental Systems Science, ETH Zurich, Zurich, 8092, Switzerland; Department of Environmental Microbiology, Eawag: Swiss Federal Institute of Aquatic Science and Technology, Dübendorf, 8600, Switzerland; Department of Environmental Systems Science, ETH Zurich, Zurich, 8092, Switzerland; Department of Environmental Microbiology, Eawag: Swiss Federal Institute of Aquatic Science and Technology, Dübendorf, 8600, Switzerland; Department of Fundamental Microbiology, University of Lausanne, Lausanne, 1015, Switzerland; Department of Fundamental Microbiology, University of Lausanne, Lausanne, 1015, Switzerland; Department of Computational Biology, University of Lausanne, Lausanne, 1015, Switzerland; Department of Biological Science, Florida State University, Tallahassee, FL, 32304, United States; Department of Fundamental Microbiology, University of Lausanne, Lausanne, 1015, Switzerland; Department of Environmental Systems Science, ETH Zurich, Zurich, 8092, Switzerland; Department of Environmental Microbiology, Eawag: Swiss Federal Institute of Aquatic Science and Technology, Dübendorf, 8600, Switzerland; Department of Fundamental Microbiology, University of Lausanne, Lausanne, 1015, Switzerland; Biozentrum, University of Basel, Basel, 4056, Switzerland; Department of Environmental Systems Science, ETH Zurich, Zurich, 8092, Switzerland; Department of Environmental Microbiology, Eawag: Swiss Federal Institute of Aquatic Science and Technology, Dübendorf, 8600, Switzerland; Department of Fundamental Microbiology, University of Lausanne, Lausanne, 1015, Switzerland; School of Biosciences, University of Sheffield, Sheffield, S10 2TN, United Kingdom; Department of Fundamental Microbiology, University of Lausanne, Lausanne, 1015, Switzerland

**Keywords:** community ecology, niche theory, trophic levels, keystone species, succession, metacommunities, neutral theory, ecological filters

## Abstract

Many microbiological outcomes are shaped by the determinants of community composition, including the factors that allow pathogens to invade healthy microbiota and the processes that maintain the diversity that underpins soil function. Community ecology provides a rich conceptual toolset to investigate patterns of coexistence that can be adapted to explain and manage these outcomes. However, these concepts have complex histories of controversy and debate that must be considered when applying them to the microbial context. Microorganisms also have distinctive characteristics that must be accounted for when studied using ideas that were originally developed to describe macroscopic ecosystems. Here, we provide a concise overview for microbiologists to five key frameworks from community ecology: Niche theory, Trophic levels, Keystone species, Succession, and Metacommunities. We discuss the historical context and controversies surrounding each framework and outline existing and potential applications to microbial systems. This work therefore provides a practical guide for microbiologists who wish to use community ecology to understand and manipulate microbial community composition.

## Introduction

Over the last three decades, genomic advances have revealed a remarkable diversity of *microorganisms (**Box 1**)*, especially prokaryotic microbes, across the planet (e.g. [1, 2]). This has led to increasing appreciation for the role that species richness and coexistence play in enhancing microbiome function [3, 4]. At the same time, it has raised fundamental questions about how microbial community diversity is maintained [5] and how community composition changes with context [6]. While we are building an increasingly detailed understanding of the biochemical relationships between microbial species [7, 8], we do not yet have a clear picture of how these translate into observed patterns of diversity and function within communities.

Box 1. Glossary of italicized terms.

**Abiotic components-** all non-living environmental factors that influence the survival and distribution of organisms within an ecosystem (e.g. temperature, water availability, pH, soil quality, nutrient levels).

**Biotic components-** the living organisms within an ecosystem or community.

**Coexistence-** long-term maintenance of different species in a given environment without extinction of community members.

**Competition-** interaction in which organisms use the same limiting resource, such as space, food, or light, which often leads to reduced growth, survival, and reproduction. Can be *interspecific* (competition between organisms of different species) or *intraspecific* (competition between organisms of the same species).

**Consumer–resource models-** mathematical frameworks used to describe reciprocal interactions between a community of consumers and a shared pool of resources. Interactions between consumers are solely mediated through competition for resources.

**Cross-feeding-** interaction in which one organism consumes compounds (e.g. secreted metabolites or publicly available products of polymer digestion) generated by another, thereby facilitating its growth and survival. Commonly found in microbial communities.

**Dispersal-** migration to and successful reproduction of individuals at sites away from their place of birth.

**Disturbance-** an event (abiotic or biotic) that causes minor to severe changes to community composition and the surrounding environment. Classic examples of disturbances include forest fires, flooding, invasive species, antibiotic treatments, and hurricanes.

**Facilitation-** interaction in which at least one species benefits and no species are harmed.

**Fitness-** a quantitative measure of the contribution of an individual to the gene pool of the next generation.

**Functional guild-** a group of organisms that acquire and process resources similarly and that have overlapping functions within an ecosystem (e.g. the same ability to break down a specific polymer). Guild members may be taxonomically distinct.

**Interactions-** relationship between organisms within an ecosystem where one species impacts the survival, growth, or reproduction of the other; reciprocal effects can occur but are not a universal feature. Types of interactions include facilitation, mutualism, commensalism, predation, and competition.

**Macroorganism-** organism that can be observed with the naked eye. Most commonly refers to multicellular species with specialized organs and tissues within the animal and plant kingdoms.

**Microorganism-** biological agent smaller than the acuity of the human eye (⪅50 μm). May include multicellular organisms (e.g. filamentous fungi), protists, bacteria, and archaea, as well as non-living phages.

**Mutualism**- interaction in which both species benefit and enhance each other’s survival, growth, or reproduction.

**Neutral dynamics-** a ‘null’ theory of ecology that assumes that all species are functionally and competitively equivalent. Community structure under neutral dynamics is purely driven by stochastic processes such as random birth, death, dispersal, and speciation.

**Niche-** the range of factors required by a species for its persistence within an ecosystem. The fundamental niche of a species denotes the range of resources and abiotic conditions that a species could use, while the realized niche consists of the actual set of resources and conditions that the organism uses, which is determined by limiting factors such as interspecific competition.

**Species-** in macroorganisms, a group of individuals capable of interbreeding to produce fertile offspring. In microorganisms, a heuristic term based on varying degrees of genetic similarity determined using full-genome sequences, operational taxonomic units, or amplicon sequence variants.

**Trade-off-** a constraint that causes an increase in a trait or function to result in the reduction of another (e.g. the limits set on total investment among different tissues by nutritional and energetic constraints).

**Top-down/bottom-up processes-** directional regulation within a trophic-structured ecosystem, whereby species occupying higher trophic levels exert control over the composition of lower trophic levels and vice versa.

A rich set of theories and tools for studying how diversity is structured and maintained already exists: community ecology [9]. This framework was originally formulated to address questions related to the composition of communities of *macroorganisms* in ecosystems such as forests and coral reefs. The observation of reproducible patterns across different ecosystem types drove the field beyond description, towards mechanistic explanations for these patterns. Modern community ecology emphasizes dynamical population processes such as birth, death, and migration as the ultimate determinants of ecosystem composition. Even apparently static communities require a balance of these processes to remain in equilibrium. Thus, to a community ecologist, the species present in an ecosystem are inseparable from the processes that cause their distributions to change, or remain stable, over time.

Community ecology is built of concepts that focus on different aspects of spatial and temporal relationships and its potential to act as a unifying framework for understanding microbial communities has been broadly acknowledged [10–13]. These previous works collectively underscore the relevance of ecological theory for interpreting microbial community structure and function, often through the lens of specific systems or conceptual themes. When applying these concepts, microbial community ecologists must consider characteristics and behaviours of microorganisms not seen in macroorganisms, such as horizontal gene transfer [14], dormancy and microbial survival strategies [15], and metabolic capabilities such as chemolithoautotrophy [16]. Moreover, microbial systems typically operate on spatial and temporal scales that are very different from those of macro-systems including rapid eco-evolutionary feedbacks [17]. This is further complicated by the lack of a clearly delineated species definition in microbial systems [18].

**Figure 1:**
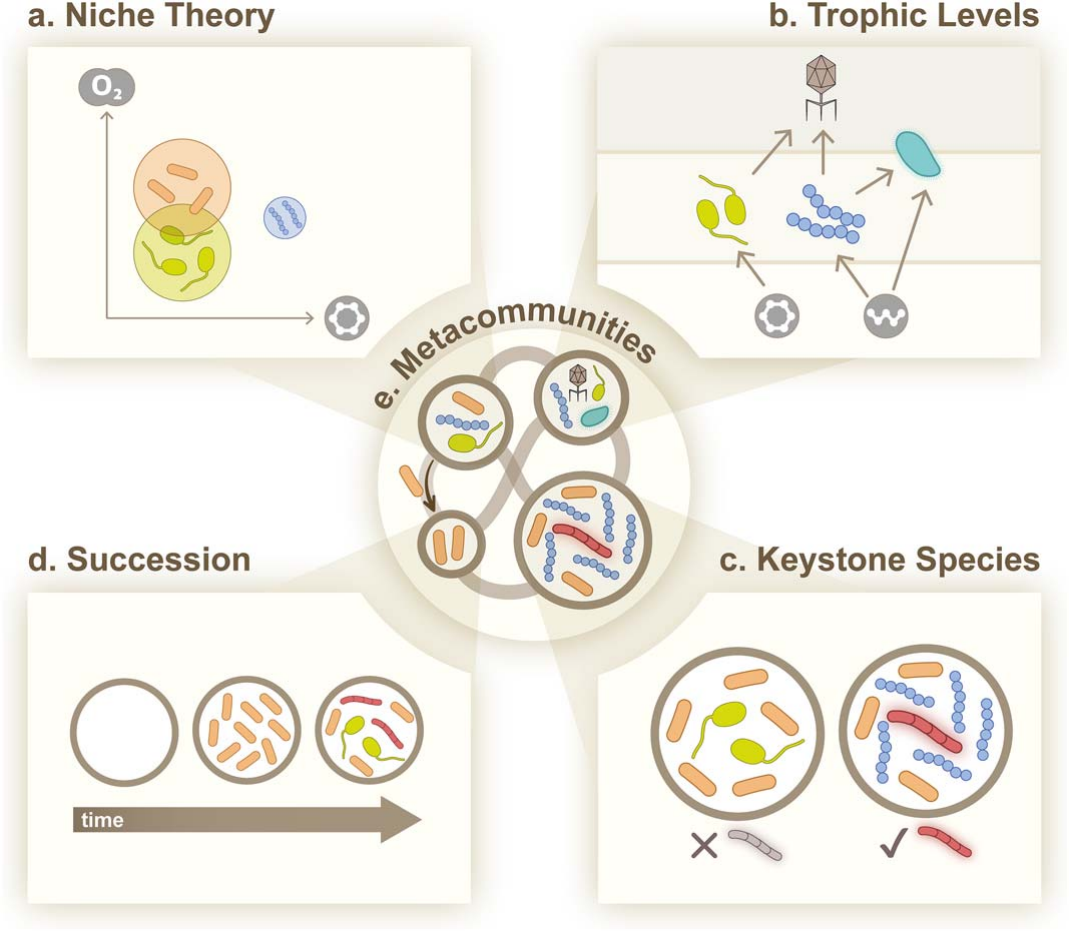
Five elements of community ecology. Niche theory (a), trophic levels (b), keystone species (c) and succession (d) are concepts applicable to communities at a given site (or ‘patch’). Metacommunities (e) are collections of such communities connected through migration of populations between patches. Axis labels in a and the bottom trophic level in b represent resources (e.g. oxygen or carbon sources), the top trophic level in b contains a phage and the segmented organism in c is a keystone species. Design and illustration by © Maria Carlos Oliveira, 2026. This content is not covered by the CC BY 4.0 licence over this publication. For permission to reuse, please contact the copyright holder.

**Figure 2:**
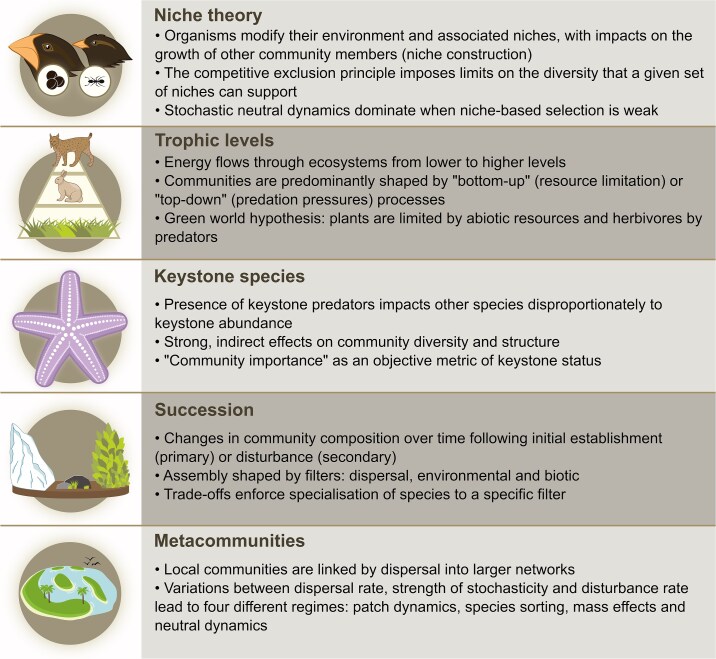
Summary of key features of community ecology concepts. Illustrations by © Maria Carlos Oliveira, 2026. Illustrations are not covered by the CC BY 4.0 licence over this publication. For permission to reuse, please contact the copyright holder.

An additional challenge to applying these concepts to microbial systems is that their meaning has historically varied depending on the system to which they are applied, leading to decades-long discussions in community ecology about their utility and correct application. In this review, we build on this foundation by synthesizing the historical development of five key concepts in community ecology, outlining how long-standing debates have shaped their current formulations. These concepts are *niche theory*, *trophic levels*, *keystone species*, *succession,* and *metacommunity theory* (Fig. 1). We focus on these five interconnected concepts as they capture complementary aspects of community ecology, from environmental constraints on species (niches), to their interactions (trophic structure and keystone effects), to their temporal dynamics (succession), and their spatial organization (metacommunities). While alternative conceptual framings have been proposed [19, 20], this group of concepts have proven useful to conservationists who face similar problems to microbiologists when managing complex communities. We further highlight characteristic patterns observed in macro-ecosystems (Fig. 2) and summarize observational, computational, and experimental approaches to study these patterns in microbial systems (Table 1). While we focus on prokaryotic microbes, these concepts have also been applied to eukaryotic microbial systems, including well-studied cases such as phytoplankton communities and protist-bacteria interactions [21, 22]. This review thus provides a practical guide for how to combine our current understanding of community ecology with the rich set of tools available to microbiologists to advance our understanding of microbial diversity and dynamics.

**Table 1:** Summary of experimental and computational approaches that can be used to test community ecology concepts.

**Manipulation of community composition**	**Phage and predator removal** (e.g. [64]) Quantify importance of top-down dynamics (trophic levels) **Single species removal** (e.g. [99]) Quantify effect of a single species on the community (keystones) Determine links in trophic chains (trophic levels) **Invasion experiments** (e.g. [45]) Evidence for utilization of separate niches in Modern Coexistence Theory (niche theory) **Dispersal manipulation** (e.g. [137]) Control of migration rates (metacommunities) **Species arrival time manipulation** (e.g. [120]) Determine priority effects (succession) **Monoculture vs. coculture** (e.g. [69]) Determine interspecific interactions and estimate interaction strengths (niche theory, keystones) Isolate trophic interaction mechanisms (trophic levels)
**Environmental manipulation**	**Spent media experiments** (e.g. [40]) Identify cross-feeding between species (niche theory) Characterize degradation chains (trophic levels) **Resource utilization screening** (e.g. [39]) Identify nutritional niches, utilization strategies and niche overlap (niche theory) **Modification of nutritional environment** (e.g. [59]) Quantify importance of bottom-up dynamics (trophic levels) Determine effect of environmental fluctuations on community composition and trophic plasticity (niche theory) **Spatial manipulation** (e.g. [138]) Control inter-patch connectivity by adjusting patch size and separation (metacommunities)
**Computational/*In situ***	**Network analyses** (e.g. [94]) Identify ecosystem hub species, interaction networks, and inter-strain relationships (keystones, trophic levels, niche theory) Fit interaction network models (e.g. generalized Lotka–Volterra) (keystones, niche theory) **Functional profiling** (e.g. [36]) Determine functions present in community members’ genomes and predict functional guilds (niche theory, trophic levels) **Machine learning methods for community dynamics** (e.g. [113]) Automated trajectory inference with abundance data (succession)

These include both established and speculative methods. The most appropriate approach for a given question will depend on the type of system being studied and the types of experiments that can be performed (e.g. purely observational studies of the human gut microbiome versus interventional studies of the mouse gut microbiome). Many of these approaches can provide evidence for multiple interconnected theories. For example, characterizing the niches of all members of a community will provide insights into how community diversity is maintained, but can also aid prediction of successional dynamics, identification of trophic levels, and determination of species sorting dynamics at the metacommunity level.

## Niche theory and neutrality

Central to community ecology is the question of how multiple populations can persist together without driving each other to extinction. An obvious solution is that organisms specialize to specific modes of life in which they do not directly compete, i.e. they occupy separate *niches* (Fig. 1a). The extensive formalization of this concept over the previous century has developed into a body of work known as niche theory (see [23]). While the niche concept itself has been refined into several interrelated sub-concepts including the establishment niche, persistence niche, realized niche, temporal niche, and fundamental niche, all niche concepts emphasize the relation between organisms and external growth-limiting factors such as the availability of nutrients, water, and space [24]. These factors may be single variables (e.g. temperature) or distributed along a continuous gradient (e.g. the size distribution of food sources). A niche can then be regarded as a specific region within this multidimensional space of environmental factors where an organism is able to grow and utilize resources.

One of the simplest examples of niche theory is the R* concept from *consumer-resource models*, which predict that multiple organisms cannot coexist when limited by a single resource because the species that can sustain itself at the lowest resource level will outcompete the others and drive them to extinction [25, 26]. Even when extended to multiple resource types and resource gradients, niche-based frameworks limit the number of coexisting species to the number of effective limiting factors in the system (also known as the competitive exclusion principle [27, 28]). This simple version of niche theory thus fails to explain the high diversity observed in ecosystems with very few limiting factors, such as tropical forests or phytoplankton. Attempts to resolve this apparent paradox has been a rich source of novel ecological concepts [22, 29].

An important advance was provided by Hubbell [30], who noted that if fitness differences between populations are small and niche-based selection is weak then slow and stochastic *neutral dynamics* become the dominant driver of extinction. As these fluctuations are purely random, they are not dependent on niche availability, releasing neutral ecosystems from the limitations of the competitive exclusion principle. Total diversity is instead determined by the balance between slow processes that increase local diversity (e.g. speciation and immigration) and the rate of stochastic extinctions [31, 32]. It is now understood that the processes governing the composition of many ecosystems lie somewhere between these extremes of purely niche-based (deterministic) and purely neutral (stochastic) [29].

Evidence for both deterministic and stochastic processes has been obtained for environmental microbial populations, as identified through sequencing-based composition analysis on *in situ* communities (Table 1). Community composition and function can be strongly correlated with temporal environmental fluctuations [33] and spatial gradients in chemical factors including salinity [34], suggesting the presence of niche-based environmental filtering. On the other hand, communities formed independently in equivalent environments often have very different compositions [35], suggesting neutral dynamics also play a strong role. A perspective that reconciles both strands of evidence is that of *functional guilds*, groups of microbes with similar functional characteristics (e.g. the set of enzymatic capabilities present in their proteomes). Importantly, phylogenetic relatedness is often a poor predictor of guild membership. For example, communities assembled in the pools of water forming on bromeliad foliage display high levels of taxonomic variation yet appear broadly similar in their functional profiles [36]. In this view, niche-based dynamics determine the assemblage of functional guilds while neutral processes select the specific guild members from within these guilds (but see [37]). The shifting role of niche-based versus neutral processes under different conditions has also been addressed using statistical approaches that partition deterministic and stochastic contributions to microbial community composition [38].

Lab-based experiments with defined communities can also be used to probe the mechanistic basis of microbial niches and the predictions of quantitative coexistence models (Table 1). As the niches of microorganisms are largely defined by their interactions with their chemical environment, there has been a strong focus on characterizing coexistence in terms of metabolism. Combining measurements of resource utilization [39] and impacts on the chemical environment imposed by microbial growth [40] allows coexistence to be explained through a complete description of niche dynamics [41, 42]. Within the context of microbial predation, the body size axis can also be an important niche determinant [43]. An alternative approach is to assess population-level dynamics without making explicit niche measurements: a key concept in macroorganismal ecology is the mutual invasibility criterion [44], which asks whether all species in a community can return to large population sizes when supressed to low numbers. This can be used as a predictor of stable coexistence and is indicative of niche-based selection [45–47]. The results of these experiments can be interpreted through the lens of Modern Coexistence Theory, which seeks to explain coexistence through phenomenological metrics capturing niche overlap and fitness differences between species [48]. However, this framework remains underapplied to microbiological data [49].

Neutral dynamics remain comparatively understudied in lab-based microcosms, possibly due to the difficulties associated with quantifying and interpreting stochastic community outcomes. Application of previously developed statistical techniques addressing this question [29, 50] to microbial communities grown under controlled conditions may provide us with deeper insights into the mechanisms that determine where communities lie on the niche/neutrality axis.

## Trophic levels

Niche theory investigates the factors that limit the growth of organisms but often over-emphasizes competition as the dominant force constraining the *realised niche*. Trophic levels provide a more realistic view in which organisms are subject to growth constraints both from ‘below’—their nutritional sources, for which there may be intra and interspecific competition—and from ‘above’—their predators. The realized niche of an organism within a trophic level is then defined by the combination of resource availability, competition, and predation.

Trophic levels have been central to ecology for almost a century [51], with Elton [52] first grouping species with similar diets and predators together. Initially, the primary use was to understand the flow of energy from primary producers to predators, leading to the iconic representation of food chains as ecological pyramids that illustrate the distribution of biomass among levels. The later delineation of *top-down processes* (predation/herbivory/parasitism and trophic cascades) and *bottom-up processes* (nutrient/resource limitation) led to the influential, if overly simplistic, ‘green world hypothesis’ that plants are limited by abiotic resources, herbivores by predators, and predators by competition for prey [53]. However, the relative importance of top-down and bottom-up constraints in community assembly and dynamics remains an ongoing debate [54, 55]. Some ecologists have even questioned the validity of discrete trophic levels altogether [56], suggesting that species’ positions within food webs are more continuous than categorical. Others suggest that variations in the importance of top-down and bottom-up forces depending on circumstances make generalities about resource control or trophic cascades impossible [57, 58]. Despite these critiques, the concept of trophic levels remains an important heuristic in ecology.

In applying trophic level concepts to microbial communities, two approaches can be taken: microbial communities can be considered a single trophic level within a wider (often macroscopic) ecosystem, or microbial communities can be themselves considered a complete ecosystem, comprised of multiple trophic levels.

### Microbial communities as a single trophic level

Microbes can be assigned to a single level in a larger food web, generally a basal level in which they consume nutrients while being predated upon by phages, protists, and metazoans (Fig. 1b). Communities in symbiotic association with metazoan hosts can also be treated as belonging to a single trophic level, albeit one that is hard to reconcile with the traditional hierarchical definition. This raises the question of whether top-down or bottom-up processes are more important for determining their composition. Bottom-up processes have dominated the thinking of microbial ecologists to date, reflected in recent studies that examine the influence of carbon source concentration, number, and type on microbial interactions and coexistence (e.g. [59]). However, less is known about top-down processes, such as protist predation [21] and phages [60–62], that can also shape microbial community structure. For example, phages have been shown to increase evenness in the abundance of competing *Pseudomonas* species [63]. Top-down and bottom-up processes in microbial communities can be controlled by independently manipulating predator activities (e.g. by suppressing a phage [64]) and resource availabilities (e.g. through glucose supplementation [65]). The relative importance of each process can then be measured by asking whether variation in resources or predation is a stronger predictor of community composition [66]. Quantification of the relative importance of these two pressures through experiments (Table 1) will give further insight into microbial community dynamics and may aid in community control by allowing prediction of whether a community will respond more strongly to changes in nutrient conditions or to the addition of new predators.

### Microbial communities as multi-trophic level systems

While this coarse-grained, functionally uniform perspective of microbial communities has been predominant in many historical analyses, modern metabolomic and genetic techniques have revealed an extraordinary diversity of metabolic relationships between near-indistinguishable microbes. Microbes possess a broad range of metabolic pathways, making them capable of photosynthesis, heterotrophy, and chemoautotrophy [16], with the latter unique to bacteria and archaea. Thus, microbial communities can themselves also be viewed as ecosystems with separate trophic levels based on species’ main source of energy. The release of metabolic by-products into the environment can also lead to syntrophic cross-feeding, mutualisms, and even co-regulation, where species influence one another’s ability to take up and process resources. For example, primary degraders can break down polymers into monomers that can be utilized by other community members [67] whose metabolism releases byproducts, such as organic acids, that can in turn benefit other community members [68]. These represent classifiable trophic interactions [8] and can be determined using culture-based experiments (Table 1). However, two factors challenge our use of the term ‘trophic level’ to describe and categorize these relationships within microbial systems: first, microbes can exhibit strong phenotypic plasticity and shift roles based on environmental context, thus making their trophic positions dynamic [69]. Second, biochemical flows through a microbial ecosystem are not necessarily linear (e.g. during nutrient cycling), violating the hierarchical assumptions of the trophic level concept. The concept of functional guilds avoids these assumptions, while still providing a level of functional grouping that can usefully simplify our picture of microbiome structure. For example, stratification of ‘succinotypes’ based on the rate of succinate consumption has been used to explore gut microbiome function and inflammatory bowel disease [70]. Metabolic niche space and functional guilds can be determined using computational methods alone or in combination with experiments (Table 1). This framework was recently used to automatically categorize the roles of heterotrophic bacteria growing on marine chitin particles, categorizing them as primary degraders, scavengers or exploiters based on genomic content. These coarse-grained categories were then related to community dynamics and overall degradation efficiency, highlighting the power of the approach for dissecting functional outcomes [60].

## Keystone species

Interactions between organisms are central to ideas of coexistence, whether they are between trophic levels (predators and prey constraining each other’s population growth) or within trophic levels (organisms with similar ecological roles competing for limited resources). When a single species has an effect through its interactions with other community members that is disproportionate to its own abundance, this is known as a keystone species [71] (Fig. 1c). The effects of keystone species are often indirect, such as when the loss of a predator causes suppression of vegetation through an increased abundance of herbivores.

While concepts similar to keystone species were described by early ecologists, the term was first coined when describing the effect of predatory *Pisaster* sea stars on the composition of tide pool communities [72, 73]. Since then, ‘keystone predation’ has been commonly used to describe increases in community diversity caused by an apex predator through regulation of interactions among prey species [74]. The use of ‘keystone’ then spread to include keystone mutualists, modifiers, herbivores, guilds, and resources [75–77]. The broad use of the term led to inconsistencies in the use and definition of ‘keystone’ that persist today [78, 79]. A wide range of both qualitative and quantitative effects have been attributed to keystone species across many different ecosystems (see [80]), and related concepts arose, such as foundation species [81] and ecosystem engineers [82]. To refocus the keystone species concept, Power and Mills proposed the ‘community importance’ of a species (the ratio of its impact on community composition in removal experiments to its abundance) as a quantitative definition [71, 80].

Keystone species have often been identified through observational studies [73]. However, these do not account for confounding factors, such as unmeasured *abiotic* variables that affect species abundances and community dynamics, and thus obscure the ecological relationships [83]. While species removal experiments provide stronger evidence, they are generally labour intensive and are challenged by scale dependence and difficulties in designing controls [78, 84, 85]. Despite this, there are well-documented examples of keystone species, including apex predators, that have advanced our understanding of species interactions and their potential effects on community dynamics [86].

Microbial communities can also contain keystone predators, such as protozoa [21], predatory metazoans [87] or bacterivorous bacteria including myxobacteria and *Bdellovibrio* [88]. Yet the concept can be difficult to apply to microbial systems, as species can rapidly adapt to changing conditions at both the physiological and genetic level, through horizontal gene transfer or rapid eco-evolutionary feedback. Further, interactions between microbial species can vary depending on the set of abiotic or *biotic* conditions present, making the identification of keystones context dependent [69, 89, 90].

Microbial keystone species have been identified based on patterns of co-occurrence [91, 92]. However, these approaches do not capture interspecies interactions or species’ ecological roles. This can be addressed computationally (Table 1) by using microbial network analyses [93, 94], through empirical dynamic modelling of microbial community composition over time [95] or by mapping interaction patterns by fitting abundance data to theoretical interaction frameworks such as the generalized Lotka–Volterra model [96]. It is important to note that the keystone species definition used in these network-based studies (i.e. a species that interacts with many other species) differs from its initial, abundance or biomass-based definition. This may be due to difficulties in obtaining absolute abundance data and inferring interactions from sequencing approaches, as well as the lack of clear trophic structure in many microbial communities. An alternative application of the keystone concept for microbial systems is to determine functional keystones whose metabolic roles are critical for maintaining a function of interest [79, 97, 98].

The prevalence and importance of keystone species in microbial communities remains an open question. A recent study by Pearl Mizrahi and coauthors [99] compared existing approaches with an alternative definition based on invasions or extinctions of other community members in response to loss of a putative keystone. Across methods, they found that such strong effects are unusual and thus conclude that keystones are rare in the investigated context. At the same time, this work highlights that we still lack a consensus on how microbial keystones should be defined, making it difficult to evaluate their prevalence across systems. Addressing this will require either the adoption of a unified definition or the development of clearly distinguished sub-concepts. One proposal is to deemphasize trophic structures and changes in abundance and instead use distinct functional and compositional definitions [98]. Additionally, computational and empirical approaches that better capture spatial and temporal contexts experienced by microbes are required to understand how fine-scale dynamics impact larger scales, allowing local processes to be linked to the large-scale patterns from which keystones are identified.

## Succession

The ecological theories above generally assume that communities are at or near equilibrium, with no long-term change in the composition of the system over time. However, most systems undergo frequent changes in environmental conditions. Rapid and disruptive environmental disturbances can open new niches, resulting in changes to both the composition of a community and the environmental context in which community members interact [100]. Succession describes compositional changes in a community over time from a disturbed starting point towards a hypothetical equilibrium (Fig. 1d).

The concept of succession was initially developed to describe the dynamics of plant communities on sand dunes [101] before being expanded to describe systems from abandoned agricultural fields [102] to tropical forests [32]. This led to considerable controversy over the underlying concepts, including the degree of determinism in successional dynamics and the validity of the concept of an inevitable ‘climax community’ to which an ecosystem in a given biome converges [103]. Our contemporary understanding of succession describes compositional changes in terms of a series of filters operating through time [104]. This view integrates long-standing discussions on successional mechanisms [100] with the closely related field of community assembly, which can lack the dynamical viewpoint of succession [105].

In this integrated perspective, an initial *dispersal*-based filter is applied to a larger, regional species pool, which determines which species will be able to disseminate to the disturbed site. An environmental filter then determines which of the arriving species will be able to grow and reproduce at that site. These two filters tend to select for organisms with similar traits, being capable of both dispersal to and establishment at the site. Local competition between established organisms, representing a final niche-based biotic filter, then reduces species similarity. The biotic filter includes interactions from other trophic levels, including predation and herbivory, as well as competition for space in sessile communities. This filter-based view means that organisms with different strategies (imposed by trade-offs) tend to dominate at different points during succession, with rapidly dispersing organisms giving way to effective colonizers and finally strong competitors [106]. Longer term, further compositional change may occur as slow environmental modifications by the community allow invasion of specialists better suited to the new conditions [107].

When using microbiomes to explore our contemporary understanding of succession (including the community assembly framework), a good starting point is to identify temporal patterns of organismal abundances in the system of interest (Table 1). This can be difficult with the high number of species typical in many microbial communities. One approach to this problem is to measure relative or absolute species abundances at different timepoints and display these data with ordination plots using eigenanalysis-based methods or distance-based methods, e.g. principal coordinate analysis or nonmetric multidimensional scaling [108]. Successional dynamics can then be identified from clear temporal sequences on these axes [109–111]. When designing such experiments it is important to clearly identify the event that acts as the beginning of the successional sequence. In contrast to the disturbance-based starting point of classic succession, many studies focus on *de novo* community assembly in a new sterile environment such as the neonatal gut [111]. Alternatively, an event such as the commingling of two previously separate communities (‘community coalescence’ [112]) may be used. A second factor to consider is the sampling timescale necessary to capture the successional dynamics, which are generally much faster in microbial than macroscopic ecosystems. The appropriate sampling timescale is system-specific, ideally being matched to the timescale of changes to the community’s composition.

Despite the growing prevalence of large sequence-based datasets, little work has been done to develop protocols for the detection of succession. We suggest using machine-learning tools, such as trajectory inference [113], to automatically infer multi-dimensional successional pathways from noisy and incomplete abundance data. This may also allow successional dynamics to be inferred when abundance data is only available from single time points for multiple communities at different stages of development.

Where a reproducible successional pattern has been observed, the next step is to identify the mechanisms that drive it. Due to the importance of metabolic *trade-offs* in the ecology of microorganisms [114, 115], many previous studies of microbial succession have attributed community changes to metabolic specialization, such as replacement of chitin degraders by cross-feeders on marine snow [116]. More broadly, successional patterns can frequently be attributed to the source of carbon within the system, with autotroph-driven communities displaying different successional patterns to purely heterotrophic systems [117]. Nonetheless, conventional succession based on a colonization-competition trade-off has also been described [118].

Successional dynamics can also be explored by manipulating the ecological filters in the ecosystem. *Priority effects* [119]—a component of the biotic filter—describe the effects of early-arriving species on the growth of later species and can be measured by artificially controlling the introduction times of different species [120] (Table 1). For instance, if changes to the environment by one species are necessary for a second to establish, this mechanism may be revealed by showing that the facilitated species fails to establish if introduced before the facilitator. Alternatively, the first species to arrive at a site may exclude all subsequent arrivals, an example of the more general phenomenon of historically contingent community assembly [19]. The effects of dispersal and growth abilities on community composition and diversity can also be manipulated in the lab and *in situ* (e.g. [121]), making microbial communities excellent systems to test successional dynamics and mechanisms.

## Metacommunities

In addition to the temporal dynamics introduced by succession, communities can also be shaped by spatial dynamics; for example, a species that goes locally extinct in one region may be reintroduced through migration from another. Metacommunity theory offers a framework to incorporate both space and time into community models by building on community ecology concepts including succession, niche theory, and trade-offs [122].

A metacommunity is a regional set of communities that occupy distinct habitat patches that are linked by dispersal of multiple interacting species [122] (Fig. 1e). In this framework, the diversity and composition of communities can be understood through the interplay between dispersal rates, competitive exclusion, and patch heterogeneity. Leibold *et al.* [122] classified metacommunities into four paradigmatic types according to whether their dynamics are governed by patch dynamics, species sorting, mass effects, or neutral dynamics. These regimes are characterized by the dominant processes that shape community composition and encompass the key concepts discussed above. For instance, in the patch dynamics regime local disturbances create heterogeneity in which successional dynamics and competition-colonization trade-offs result in differences in community composition among patches. This is in opposition to the neutral regime in which species have small or zero fitness differences and species distributions are influenced solely by stochastic events. However, it is very rare that an ecosystem is described by only one of these four regimes. Therefore, while a useful heuristic, the utility of these four metacommunity paradigms has since been questioned as natural communities rarely fit neatly into just one [123, 124]. More recent work has focused on how individual species and patches contribute to overall metacommunity properties and diversity using analytical tools such as joint species distribution models [124].

The basic concept of metacommunity theory may initially appear highly applicable to microbial communities, as dispersal, competition/colonization trade-offs, and local habitats with patch dynamics are common features of microbial habitats. Further, empirical tests of the metacommunity framework have been outlined with both macro and micro systems in mind [125, 126]. However, several features of microbial communities often make the original metacommunity framework difficult to apply. Firstly, it can be difficult to determine patch size in order to assess the appropriate scale for spatial sampling. Natural habitat patches can vary in their characteristics [126]: patches may have boundaries but be ephemeral (e.g. marine snow particles) or be more permanent but with indistinct boundaries (e.g. zones along plant roots). Microbial communities can also exhibit hierarchal structures with different spatial scales creating different kinds of patches and potentially patches within patches (e.g. the microbiome of the oral and nasal cavities within the human body, [127]). The spatial scales at which microbes are interacting within these patches adds further complexity, and the importance of short-range versus long-range interactions is an ongoing topic of research [128–130]. In these cases, rather than the four paradigms, microbial community dynamics may be better described directly by the birth, death, and dispersal processes that are responsible for the changes in both species density and the local environment. Meta-ecosystem theory, which expands on classic metacommunity theory to describe spatiotemporal flows of energy and resources [131, 132], may be also better suited to capture microbial community dynamics provided appropriate measurements can be made to parameterize the models.

Recent work has applied the metacommunity and meta-ecosystem frameworks to microbial communities. For example, Miller and Bohannon [133] expanded the metacommunity theory to incorporate bacterial growth during dispersal between patches. Other studies [134–136] have also investigated ecosystem flux and metacommunity dynamics in multi-trophic communities of plankton and bacteria. However, these frameworks remain underutilized with relatively few studies applying these concepts to microbial systems. Microbial communities offer highly manipulable systems in which the mechanistic processes driving metacommunity dynamics can be controlled (Table 1). Dispersal, key to metacommunity theory, can easily be adjusted with appropriately designed transfers of cultures between microcosms (allowing testing of metacommunity dynamics, [137]) while chemostats and microfluidic chips allow for the microorganisms’ feedback with the environment to be controlled (allowing the testing of meta-ecosystem predictions). Microfluidic chips, artificial soil, and droplet-based experiments [118, 138–140] also create semi-realistic spatial habitats that allow for the effects of landscape heterogeneity and fragmentation to be compared with theoretical predictions [141]. Microbial systems are therefore particularly well suited for testing metacommunity and meta-ecosystem theories, and, in turn, these frameworks can further advance our understanding of the mechanisms and processes driving microbial species distributions and densities at the single patch and regional scales.

## Microbial community ecology: new insights from old ideas

Microbiology has entered an era of unprecedented experimental resolution. High-throughput techniques allow us to probe communities at fine temporal and spatial scales, abiotic conditions can be precisely manipulated and monitored, and microscopy enables real-time observation of the physiological states of interacting microorganisms at the single-cell level. Just as modern genome sequencing has revealed intricacies of evolutionary relationships, these advances allow us to link concepts in community ecology to underlying mechanisms in ways that were previously unattainable. As data collection continues to accelerate, the need for unifying conceptual frameworks through which these measurements can be interpreted will only grow.

Nonetheless, several ecological topics that are widely studied among macroorganisms remain poorly explored at the microscopic level. Unlike associations between animals, many microbial behaviours and interactions remain poorly understood as they are mediated through biochemical processes that cannot be directly observed. As a result, these are often inferred indirectly from their effects on growth, survival or community dynamics. However, continuing improvements to labelling and imaging techniques promise to open the field of microbial behavioural ecology in the near future.

Understanding the historical development of such ecological concepts will help us to understand their nuances. As highlighted by Trubl and Probst [142], further progress depends not only on developing a refined set of concepts, but also on establishing standards in terminology to create a common language. Without this, new concepts can obscure underlying mechanisms by blurring the lines between existing ideas. Such standards should also be prioritized while developing microbe-specific concepts, such as the black queen hypothesis [143] and adaptive suicide [144].

Ultimately, the value of these concepts lies in how they structure our research. For example, trophic levels and keystone species provide a starting point for understanding the role of phages in structuring bacterial communities [61]. Community ecology may also provide conceptual tools to help us understand the role of scale in microbial ecosystems [145]: at the largest scales, microbial communities are incredibly ‘species’ rich, often surpassing the community diversity observed in macroscopic systems. However, this coarse-grained sampling neglects the microscopic scale of microbial interactions and the heterogeneity of the micro-environment. Ecological theory can help us to identify the appropriate scales that must be sampled to capture a particular phenomenon. In return, microbial systems can advance classic ecological frameworks by offering ideal experimental settings to test their predictions and assess their generality. Both microbiology and community ecology will continue to advance from this ongoing exchange, providing a clearer picture of the processes that determine community composition and function in natural ecosystems.

## Data Availability

Data sharing is not applicable to this article as no datasets were generated or analysed during the current study.
